# Rendering neuronal state equations compatible with the principle of stationary action

**DOI:** 10.1186/s13408-021-00108-0

**Published:** 2021-08-12

**Authors:** Erik D. Fagerholm, W. M. C. Foulkes, Karl J. Friston, Rosalyn J. Moran, Robert Leech

**Affiliations:** 1 Department of Neuroimaging, King’s College London, London, UK; 2 Department of Physics, Imperial College London, London, UK; 3 Wellcome Centre for Human Neuroimaging, University College London, London, UK

**Keywords:** Stationary action, Lagrangian, Computational neuroscience, Neural state equations

## Abstract

The principle of stationary action is a cornerstone of modern physics, providing a powerful framework for investigating dynamical systems found in classical mechanics through to quantum field theory. However, computational neuroscience, despite its heavy reliance on concepts in physics, is anomalous in this regard as its main equations of motion are not compatible with a Lagrangian formulation and hence with the principle of stationary action. Taking the Dynamic Causal Modelling (DCM) neuronal state equation as an instructive archetype of the first-order linear differential equations commonly found in computational neuroscience, we show that it is possible to make certain modifications to this equation to render it compatible with the principle of stationary action. Specifically, we show that a Lagrangian formulation of the DCM neuronal state equation is facilitated using a complex dependent variable, an oscillatory solution, and a Hermitian intrinsic connectivity matrix. We first demonstrate proof of principle by using Bayesian model inversion to show that both the original and modified models can be correctly identified via *in silico* data generated directly from their respective equations of motion. We then provide motivation for adopting the modified models in neuroscience by using three different types of publicly available *in vivo* neuroimaging datasets, together with open source MATLAB code, to show that the modified (oscillatory) model provides a more parsimonious explanation for some of these empirical timeseries. It is our hope that this work will, in combination with existing techniques, allow people to explore the symmetries and associated conservation laws within neural systems – and to exploit the computational expediency facilitated by direct variational techniques.

## Introduction

Virtually all of modern physics has been formulated in terms of the principle of stationary action, from Maxwell’s equations in electromagnetism [1], to the Einstein field equations in the general theory of relativity [2], through to the Dirac equation in quantum mechanics [3]. This approach has many advantages. Firstly, coupled sets of equations of motion can be described in terms of a single Lagrangian function, allowing for a parsimonious unified mathematical framework – as was famously demonstrated via the single Lagrangian formulation of the entire standard model of particle physics [4]. Lagrangian formulations also allow for otherwise inaccessible insights into dynamical systems, given that they uncover otherwise hidden symmetries and associated conservation laws [5]. Furthermore, direct variational techniques present potentially important applications in the analysis of dynamical systems, given that they allow for equations of motion to be bypassed entirely [6, 7] – therefore greatly expediting the execution times of forward models.

In classical physics, dynamical systems are framed in terms of equations of motion describing quantities such as position, velocity, and acceleration. On the other hand, the equations of motion used in computational neuroscience refer to more abstract quantities, such as membrane potentials, firing rates, and macroscopic neuronal activity [8]. Variational techniques have been used in conjunction with neural networks, regarding the construction of path integral representations of stochastic dynamics. These techniques elucidate the systematic corrections to mean-field results due to stochasticity, and allow the calculation of moments of activity, as well as the application of renormalization group methods in critical states [9–12]. These formulations have also been shown to be applicable to disordered systems, for example neuronal networks with randomly drawn connectivity [13].

This paper comprises three sections.

In the first section, using the Dynamic Causal Modelling (DCM) neuronal state equation [14] as an archetype of the first-order linear state equations used in computational neuroscience, we show that it is possible to make certain modifications to its mathematical form that allow for a Lagrangian formulation. Specifically, three such modifications are found to be necessary: (a) the dependent variable must be complex; (b) the left-hand side of the state equation must be multiplied by the imaginary unit *i* – a modification that fundamentally alters the model by changing the solutions from non-oscillatory to oscillatory; and (c) the intrinsic coupling matrix must be Hermitian. We stress that the original (unmodified) state equation cannot be recovered from the Lagrangian formulation – only the modified complex, oscillatory form allows for compatibility with the principle of stationary action.

In the second section, we provide proof of principle by demonstrating that both the original (non-complex, non-oscillatory) and modified (complex, oscillatory) neuronal state equations can be correctly identified. To do this, we generate two sets of synthetic data – one using the original model and one using the modified equation of motion. For each of these two datasets, we then use Bayesian model inversion to evaluate the respective variational free energy (model evidence); using both the original, as well as the modified equations of motion. We find that the variational free energy correctly assigns the original data to the equation of motion that generated those data – thus demonstrating that this technique can disambiguate between the genesis of these data: i.e., that the implicit models are identifiable – and that the (complex, oscillatory) modification has a material effect on observed dynamics.

In the third section, we show that the modified equation of motion provides higher model evidence in publicly available datasets obtained using three different neuroimaging techniques – electroencephalography (EEG), functional near-infrared spectroscopy (fNIRS), and electrocorticography (ECoG). These numerical analyses underwrite the generalisability of – and provide empirical motivation for – adopting the modified state equation in certain cases. We provide open-source MATLAB code that reproduces the results presented in this paper – both for the synthetic and experimental datasets in question.

## Main text

### The DCM neuronal state equation

A generic nonlinear dynamical system can be expressed in terms of a Taylor series expansion [15], which in its simplest form, for the *i*th region, is expressed as the linear DCM neuronal state equation:

1 $$\begin{aligned}& \dot{z}_{i} ( t ) = \sum_{j} A_{ij} z_{j} ( t ) + \sum_{j} C_{ij} v_{j} ( t ) + \omega _{i}^{ ( z )} ( t ), \end{aligned}$$

where *z* represents the state of the system in question; *A* is the intrinsic coupling matrix; *C* is the extrinsic input matrix; $v =u+ \omega ^{ ( v )}$, where $\omega ^{ ( v )}$ is a noise term describing random, non-Markovian fluctuations on external perturbations *u*; and $\omega ^{ ( z )}$ is a noise term describing random, non-Markovian fluctuations on *z* [16]. Using Eq. (1), we can obtain estimates of latent model parameters in the presence of noise on states via Bayesian model inversion. These model parameters include the ways in which the system in question is connected, both intrinsically and extrinsically, with the surrounding environment.

### A failed attempt using real variables

We will now show why complex variables are necessary when casting Eq. (1) – and by extension any first-order linear differential equation – in the form of a Lagrangian. We will prove this first by contradiction – i.e., we begin by attempting to use real variables and demonstrate that this leads to a non-unique solution. If we insist on using real variables then (as shown below) we must assume that *v* and *ω* are constant in time, in which case we can re-write Eq. (1) as follows:

2 $$\begin{aligned}& \dot{z}_{i} ( t ) = \sum_{j} A_{ij} z_{j} ( t ) + d_{i}, \end{aligned}$$

where $d_{i} = \sum_{j} C_{ij} v_{j} + \omega _{i}^{ ( z )}$ is a constant.

Differentiating Eq. (2) with respect to time we obtain $\ddot{z}_{i} ( t ) = \sum_{j} A_{ij} \dot{z}_{j} ( t )$ which, together with Eq. (2), gives

3 $$\begin{aligned}& \ddot{z}_{i} ( t ) = \sum_{j} E_{ij} z_{j} ( t ) + f_{i}, \end{aligned}$$

where $E= A^{2}$ and $f_{i} = \sum_{j} A_{ij} d_{j}$.

We then note that, if the *E* matrix is symmetric, Eq. (3) can be viewed as the Euler–Lagrange equation:

4 $$\begin{aligned}& \frac{d}{dt} \biggl[ \frac{\partial \mathcal{L}}{\partial \dot{z}_{i}} \biggr] = \frac{\partial \mathcal{L}}{\partial z_{i}}, \end{aligned}$$

for the Lagrangian given by

5 $$\begin{aligned}& \mathcal{L}= \sum_{j} \biggl( \frac{1}{2} \dot{z}_{j}^{2} + f_{j} z_{j} \biggr) + \frac{1}{2} \sum_{j,k} E_{jk} z_{j} z_{k}, \end{aligned}$$

thereby giving a Lagrangian description of Eq. (3).

However, although any solution of the first-order Eq. (2) is also a solution of the second-order Euler–Lagrange equation Eq. (4), the reverse is not true: the second-order Euler–Lagrange equation Eq. (4) also has other solutions that are not valid solutions of the original DCM recovery model Eq. (2). To illustrate the problem, consider a simple example with only one region. The DCM equation simplifies to $\dot{z} =Az+d$, the general solution of which is $z= k_{1} e^{At} - \frac{d}{A}$, where $k_{1}$ is an arbitrary constant of integration. The general solution of the corresponding Euler–Lagrange equation, $\ddot{z} = A^{2} z+Ad$, is $z= k_{1} e^{At} + k_{2} e^{-At} - \frac{d}{A}$, where $k_{1}$ and $k_{2}$ are both arbitrary constants. This is just one example of a general problem: if one differentiates a first-order differential equation, the general solution of the resulting second-order ordinary differential equation depends on two arbitrary constants – not one. Every solution of the first-order equation must also be a solution of the second-order equation, but most of the solutions of the second-order equation do not satisfy the first-order equation. Therefore, it is not possible to cast the neuronal state equation Eq. (1) in the form of a Lagrangian in a way that allows either for the modelling of time-dependent external inputs and noise, or for unique recovery via the principle of stationary action.

### The complex oscillatory equation of motion

We will now demonstrate that it is possible to cast the neuronal state equation Eq. (1) in the form of a Lagrangian as long as the following three conditions are met: (a) the dependent variable *z* is complex; (b) the left-hand side is multiplied by the imaginary unit *i* – thus rendering the solutions oscillatory; and (c) the *A* and *C* matrices in Eq. (1) are Hermitian. This modified neuronal state equation is written as follows:

6 $$\begin{aligned}& i \dot{z}_{i} = \sum_{j} A_{ij} z_{j} + \sum_{j} C_{ij} v_{j} + \omega _{i}^{ ( z )}. \end{aligned}$$

### The neuronal state Lagrangian

We are now able to put forward the central proposition of this paper, which is that that Eq. (6) can be derived from the following Lagrangian:

7 $$\begin{aligned}& \mathcal{L}= \frac{i}{2} \sum_{j} \bigl( z_{j}^{*} \dot{z}_{j} - z_{j} \dot{z}_{j}^{*} \bigr) - \sum_{j,k} \bigl( z_{j}^{*} A_{jk} z_{k} + z_{j}^{*} C_{jk} v_{k} + v_{j} C_{jk} z_{k} \bigr) - \sum _{j} \omega _{j}^{ ( z )} \bigl( z_{j} + z_{j}^{*} \bigr), \end{aligned}$$

where we use star notation to indicate complex conjugation and have assumed that the external perturbations $v_{j}$ and noises $\omega _{j}^{(z)}$ are real and, as the *A* and *C* matrices are Hermitian, the Lagrangian is also real.

We will show that this proposed Lagrangian is correct by verifying that the original equation of motion Eq. (6) can be recovered from Eq. (7) via the principle of stationary action. To do this, we temporarily proceed under the assumption that the variables *z*, $z^{*}$, *ż*, and $\dot{z}^{*}$ are independent of one another – it will become clear below why this is a valid assumption. We can then write the general variation of $\mathcal{L} ( z, \dot{z,} z^{*}, \dot{z}^{*} )$ as follows:

8 $$\begin{aligned}& \delta \mathcal{L} = \frac{\partial \mathcal{L}}{\partial z} \delta z+ \frac{\partial \mathcal{L}}{\partial \dot{z}} \delta \dot{z} + \frac{\partial \mathcal{L}}{\partial z^{*}} \delta z^{*} + \frac{\partial \mathcal{L}}{\partial \dot{z}^{*}} \delta \dot{z}^{*} . \end{aligned}$$

Since the values of *z*, *ż*, $z^{*}$, $\dot{z}^{*}$ and their variations *δz*, *δż*, $\delta z^{*}$, $\delta \dot{z}^{*}$ are all arbitrary, this equation must also hold when we add restrictions by insisting that: (a) $z^{*}$ must be equal to the complex conjugate of *z* (and hence $\delta z^{*}$ must be equal to the complex conjugate of *δz*); and (b) that *ż* is the derivative of *z* (and hence *δż* is the time derivative of *δz*). In other words, although we begin by treating *z*, *ż*, $z^{*}$ and $\dot{z}^{*}$ as independent variables, Eq. (8) must also hold when $z^{*}$ is the complex conjugate of *z* and *ż* is the time derivative of *z*. Assuming this to be the case from now on, the Lagrange equations of motion are derived in the standard way by looking for functions (“paths”) $z(t)$ that render the action $S [ z(t) ] = \int _{t_{i}}^{t_{f}} \mathcal{L} ( z ( t ), \dot{z} ( t ), z^{*} ( t ), \dot{z}^{*} ( t ) )\,dt$ stationary. We consider two related variations of $z(t)$ given by

9 $$\begin{aligned}& \begin{aligned}& \delta z_{1} (t) = \delta \eta (t) \quad \rightarrow \quad \delta z_{1}^{*} (t) = \delta \eta ^{*} (t), \\ & \delta z_{2} ( t ) = i\delta \eta ( t ) \quad \rightarrow\quad \delta z_{2}^{*} ( t ) =- i\delta \eta ^{*} (t), \end{aligned} \end{aligned}$$

where $\delta\eta (t)$ is any differentiable complex function of time vanishing at the initial time $t_{i}$ and the final time $t_{f}$. Using Eq. (8), together with the two variations in Eq. (9) (both of which are general because $\delta \eta (t)$ is a general variation), the principle of stationary action tells us that:

10 $$\begin{aligned}& \begin{aligned}&\delta S_{1} = \int _{t_{i}}^{t_{f}} \delta \mathcal{L}_{1}\,dt = \int _{t_{i}}^{t_{f}} \biggl( \frac{\partial \mathcal{L}}{ \partial z} \delta \eta + \frac{\partial \mathcal{L}}{\partial \dot{z}} \delta \dot{\eta } + \frac{\partial \mathcal{L}}{\partial z^{*}} \delta \eta ^{*} + \frac{\partial \mathcal{L}}{ \partial \dot{z}^{*}} \delta \dot{\eta }^{*} \biggr)\,dt=0, \\ & \delta S_{2} = \int _{t_{i}}^{t_{f}} \delta \mathcal{L}_{2}\,dt = i \int _{t_{i}}^{t_{f}} \biggl( \frac{\partial \mathcal{L}}{\partial z} \delta \eta + \frac{\partial \mathcal{L}}{ \partial \dot{z}} \delta \dot{\eta } - \frac{\partial \mathcal{L}}{\partial z^{*}} \delta \eta ^{*} - \frac{\partial \mathcal{L}}{\partial \dot{z}^{*}} \delta \dot{\eta }^{*} \biggr)\,dt=0, \end{aligned} \end{aligned}$$

where we divide the second equation by *i* and add/subtract it to/from the first to give

11 $$\begin{aligned}& \begin{aligned}& \int _{t_{i}}^{t_{f}} \biggl( \frac{\partial \mathcal{L}}{\partial z} \delta \eta + \frac{\partial \mathcal{L}}{\partial \dot{z}} \delta \dot{\eta } \biggr)\,dt =0, \\ & \int _{t_{i}}^{t_{f}} \biggl( \frac{\partial \mathcal{L}}{\partial z^{*}} \delta \eta ^{*} + \frac{\partial \mathcal{L}}{\partial \dot{z}^{*}} \delta \dot{\eta }^{*} \biggr)\,dt =0. \end{aligned} \end{aligned}$$

Finally, we integrate the second terms in Eq. (11) by parts, noting that the boundary terms vanish because $\delta \eta =0$ at $t_{i}$ and $t_{f}$, to obtain

12 $$\begin{aligned}& \begin{aligned}& \int _{t_{i}}^{t_{f}} \biggl( \frac{\partial \mathcal{L}}{\partial z} - \frac{d}{dt} \biggl( \frac{\partial \mathcal{L}}{\partial \dot{z}} \biggr) \biggr) \delta \eta \,dt =0, \\ & \int _{t_{i}}^{t_{f}} \biggl( \frac{\partial \mathcal{L}}{\partial z^{*}} - \frac{d}{dt} \biggl( \frac{\partial \mathcal{L}}{\partial \dot{z}^{*}} \biggr) \biggr) \delta \eta ^{*}\,dt =0. \end{aligned} \end{aligned}$$

Since *δη* is arbitrary, the Euler–Lagrange equations

13 $$\begin{aligned}& \begin{aligned}& \frac{\partial \mathcal{L}}{\partial z} - \frac{d}{dt} \biggl( \frac{\partial \mathcal{L}}{\partial \dot{z}} \biggr) =0, \\ & \frac{\partial \mathcal{L}}{\partial z^{*}} - \frac{d}{dt} \biggl( \frac{\partial \mathcal{L}}{\partial \dot{z}^{*}} \biggr) =0, \end{aligned} \end{aligned}$$

follow directly from Eq. (12).

Evaluating the necessary derivatives of our proposed Lagrangian in Eq. (7):

14 $$\begin{aligned}& \begin{aligned}& \frac{\partial \mathcal{L}}{\partial z_{i}^{*}} = \frac{i}{2} \dot{z}_{i} - \omega _{i}^{ ( z )} - \sum_{j} A_{ij} z_{j} - \sum_{j} C_{ij} v_{j} , \\ & \frac{\partial \mathcal{L}}{\partial z_{i}} = - \frac{i}{2} \dot{z}_{i}^{*} - \omega _{i}^{ ( z )} - \sum_{j} z_{j}^{*} A_{ji} - \sum _{j} v_{j} C_{ji}, \\ & \frac{d}{dt} \biggl[ \frac{\partial \mathcal{L}}{\partial \dot{z}_{i}^{*}} \biggr] =- \frac{i}{2} \dot{z}_{i}, \\ & \frac{d}{dt} \biggl[ \frac{\partial \mathcal{L}}{\partial \dot{z}_{i}} \biggr] = \frac{i}{2} \dot{z}_{i}^{*}, \end{aligned} \end{aligned}$$

shows that the corresponding Euler–Lagrange equations are

15 $$\begin{aligned}& \begin{aligned}& i \dot{z}_{i} = \sum_{j} A_{ij} z_{j} + \sum_{j} C_{ij} v_{j} + \omega _{i}^{ ( z )}, \\ & - i \dot{z}_{i}^{*} = \sum_{j} z_{j}^{*} A_{ji} + \sum _{j} v_{j} C_{ji} + \omega _{i}^{ ( z )}, \end{aligned} \end{aligned}$$

i.e., we recover the complex oscillatory DCM neuronal state equation Eq. (6) and its adjoint. Because the *A* and *C* matrices are Hermitian, these two equations are complex conjugates of each other – i.e., whenever one is true, so is the other and we do not need to solve them separately. It is for this reason that we must include an imaginary unit *i* in the Lagrangian formulation – the addition flips the signs of the $\frac{\partial }{\partial t}$ terms in the complex conjugate of the Lagrangian in Eq. (7) and hence renders the derivatives with respect to *z* and $z^{*}$ complex conjugates of one another. Note that, unlike the example case using real variables in Eq. (2) through Eq. (5), the use of complex variables allows for the modified state equation Eq. (6) to be uniquely recovered in a way that allows for both time-dependent external inputs and noise terms.

### The neuronal state Hamiltonian

The Hamiltonian $\mathcal{H}$ is related to the Lagrangian via the Legendre transform: $\mathcal{H}= \sum_{k} \frac{\partial \mathcal{L}}{\partial \dot{z}_{k}} z_{k} -\mathcal{L}$, where, in the case of Eq. (7), the summation is taken over the two variables *z* and $z^{*}$, such that

16 $$\begin{aligned} \mathcal{H} = &\sum_{k} \biggl( \frac{\partial \mathcal{L}}{\partial \dot{z}_{k}} \dot{z}_{k} + \frac{\partial \mathcal{L}}{\partial \dot{z}_{k}^{*}} \dot{z}_{k}^{*} \biggr) -\mathcal{L} \\ =& \sum_{j,k} \bigl( z_{j}^{*} A_{jk} z_{k} + z_{j}^{*} C_{jk} v_{k} + v_{j} C_{jk} z_{k} \bigr) + \sum_{j} \omega _{j}^{ ( z )} \bigl( z_{j} + z_{j}^{*} \bigr). \end{aligned}$$

Note that the neuronal system is influenced by time-dependent external perturbations *v* and noise *ω*. The time-translation invariance of the Lagrangian that leads, via Noether’s theorem [5], to the principle of energy conservation therefore does not hold and the value of the Hamiltonian function (energy) is not conserved. However, we can consider a non-dissipative version of the neuronal state equation and its adjoint:

17 $$\begin{aligned}& i \dot{z}_{i} = \sum_{j} A_{ij} z_{j}, \end{aligned}$$

18 $$\begin{aligned}& -i \dot{z}_{i}^{*} = \sum_{j} z_{j}^{*} A_{ji}, \end{aligned}$$

for which, by comparison with Eqs. (7) and (16), the associated Lagrangian and Hamiltonian are given by

19 $$\begin{aligned}& \begin{aligned}& \mathcal{L}= \frac{i}{2} \sum_{j} \bigl( z_{j}^{*} \dot{z}_{j} - z_{j} \dot{z}_{j}^{*} \bigr) - \sum_{j, k} z_{j}^{*} A_{jk} z_{k}, \\ & \mathcal{H}= \sum_{j,k} z_{j}^{*} A_{jk} z_{k}. \end{aligned} \end{aligned}$$

To show that the Hamiltonian is indeed conserved, we differentiate it in time as follows:

20 $$\begin{aligned}& \dot{\mathcal{H}} = \frac{d}{dt} \biggl( \sum_{j,k} z_{j}^{*} A_{jk} z_{k} \biggr) = \sum _{j, k} \dot{z}_{j}^{*} A_{jk} z_{k} + \sum_{j, k} z_{j}^{*} A_{jk} \dot{z}_{k} \end{aligned}$$

which, using Eqs. (13), (17), and (18), reads

21 $$\begin{aligned}& \dot{\mathcal{H}} =i \sum_{j} \dot{z}_{j}^{*} \dot{z}_{j} -i \sum _{k} \dot{z}_{k}^{*} \dot{z}_{k} = 0 . \end{aligned}$$

i.e., energy does not change in time, meaning that it is conserved by virtue of time translational invariance in the underlying equations of motion Eqs. (17) and (18).

### Simulations

**Figure 1:**
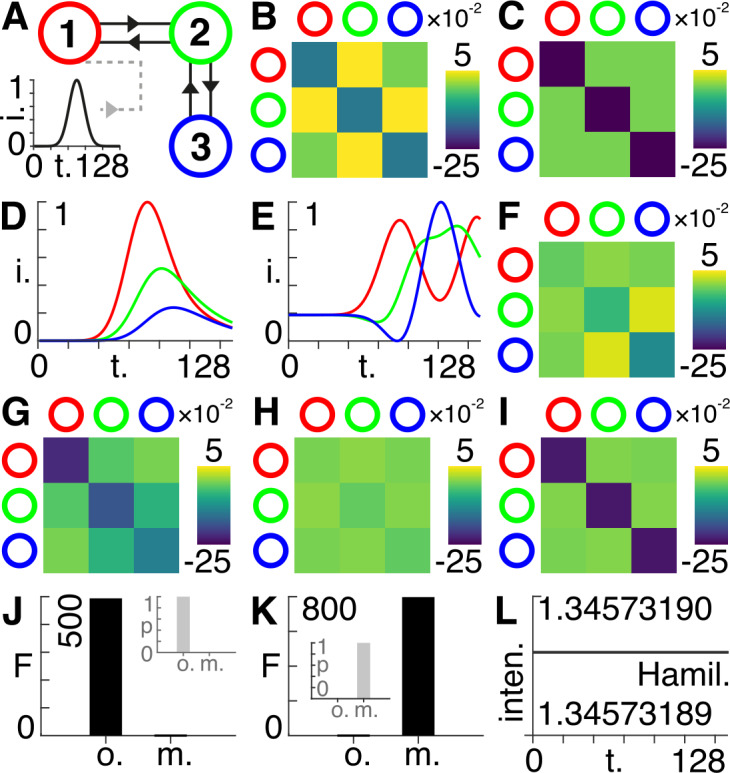
Simulations. (**A**) Three bi-directed nodes shown by the red, green, and blue circles. The first (red) node receives an exogenous input in the form of a Gaussian bump function (inset bottom left), shown as normalized intensity (*i*.) vs. time steps (*t*.). (**B**) The intrinsic coupling matrix with values corresponding to the colour bar shown on the right. Rows and columns correspond to the nodes shown in (**A**) via the colours shown on the outside of the matrix. (**C**) The priors set for the intrinsic coupling matrix had values corresponding to the colour bar shown on the right. Rows and columns correspond to the nodes shown in (**A**) via the colours shown on the outside of the matrix. (**D**) The synthetic data generated by the original state equation, shown as normalized intensity (*i*.) vs. time steps (*t*.), with colours corresponding to those of the nodes shown in (**A**). (**E**) The synthetic data generated by the modified state equation, shown as normalized intensity (*i*.) vs. time steps (*t*.), with colours corresponding to those of the nodes shown in (**A**). (**F**) The posterior estimates for the intrinsic coupling matrix following Bayesian model inversion with the original model for the data generated by the original state equation in (**D**), with values corresponding to the colour bar shown on the right. Rows and columns correspond to the nodes shown in (**A**) via the colours shown on the outside of the matrix. (**G**) The posterior estimates for the intrinsic coupling matrix following Bayesian model inversion with the modified model for the data generated by the original state equation in (**D**), with values corresponding to the colour bar shown on the right. Rows and columns correspond to the nodes shown in (**A**) via the colours shown on the outside of the matrix. (**H**) The posterior estimates for the intrinsic coupling matrix following Bayesian model inversion with the original model for the data generated by the modified state equation in (**E**), with values corresponding to the colour bar shown on the right. Rows and columns correspond to the nodes shown in (**A**) via the colours shown on the outside of the matrix. (**I**) The posterior estimates for the intrinsic coupling matrix following Bayesian model inversion with the modified model for the data generated by the modified state equation in (**E**), with values corresponding to the colour bar shown on the right. Rows and columns correspond to the nodes shown in (**A**) via the colours shown on the outside of the matrix. (**J**) Approximate lower bound log model evidence given by the free energy (*F*) following Bayesian model inversion for the original (*o*.) and modified (*m*.) models using the data generated by the original model in (**D**). Probabilities (*p*) derived from the log evidence are shown in the inset top right. (**K**) Approximate lower bound log model evidence given by the free energy (*F*) following Bayesian model inversion for the original (*o*.) and modified (*m*.) models using the data generated by the modified model in (**E**). Probabilities (*p*) derived from the log evidence are shown in the inset left. (**L**) The intensity (inten.) at every point in time for the non-dissipative form of the modified state equation, i.e., excluding external driving inputs and noise, using the posteriors from (**I**), using the Hamiltonian (Hamil.) as the observer equation.

Here, we consider a network consisting of three connected nodes, with the first node receiving an external driving input (Fig. 1(A)). This external input provides a Gaussian ‘bump’ function of peristimulus time (Fig. 1(A) – inset). The network is intrinsically connected with symmetric off-diagonal elements and negative leading diagonal components. This initializes the system with stable dynamics because the eigenvalues of the Jacobian all have negative real components (Fig. 1(B)). We then specify the priors of the implicit coupling matrix by setting all off-diagonal coupling parameters to zero and by setting the leading diagonal elements to $- {1} / {4}$ (Fig. 1(C)) – see the accompanying code for all priors and settings. We then use the original state equation Eq. (1) to run the model forward and generate synthetic data (Fig. 1(D)). Next, we change the dependent variable so that it comprises two components – one real $\operatorname{Re} ( t )$ and one imaginary $\operatorname{Im} ( t )$ – and multiply the left-hand side by the imaginary unit *i*, such that we now deal with the modified state equation Eq. (6) written as

22 $$\begin{aligned}& i \biggl( \frac{d}{dt} ( \operatorname{Re}_{i} ) +i \frac{d}{dt} ( \operatorname{Im}_{i} ) \biggr) = \sum _{j} A_{ij} ( \operatorname{Re}_{j} +i \operatorname{Im}_{j} ) + \sum_{j} C_{ij} v_{j} + \omega _{i}^{ ( z )} . \end{aligned}$$

Here, we equate the real and imaginary components gives the following two equations:

23 $$\begin{aligned}& \begin{aligned}& \frac{d}{dt} ( \operatorname{Im}_{i} ) =- \biggl( \sum_{j} A_{ij} \operatorname{Re}_{j} + \sum_{j} C_{ij} v_{j} + \omega _{i}^{ ( \operatorname{Im} )} \biggr), \\ & \frac{d}{dt} ( \operatorname{Re}_{i} ) = \sum _{j} A_{ij} \operatorname{Im}_{i}, \end{aligned} \end{aligned}$$

where we use an observation equation that retains the real component (see accompanying code) to generate synthetic data via the modified model (Fig. 1(E)).

We then performed four separate model inversions – using dynamic expectation maximization (DEM) [17] – to retrieve posterior estimates of the intrinsic connectivities for: (a) the original data with the original model (Fig. 1(F)); (b) the original data with the modified model (Fig. 1(G)); (c) the modified data with the original model (Fig. 1(H)); and (d) the modified data with the modified model (Fig. 1(I)). Note that Fig. 1(I) shows that the priors in Fig. 1(C) are closely recovered – however, not perfectly, as seen by running the accompanying code. We can now compare the variational free energies (and associated probabilities) for (a) and (b) – showing that the original data is better fitted using the original model (Fig. 1(J)). Similarly, we can compare the variational free energies (and associated probabilities) for (c) and (d) – showing that the modified data is better explained by the modified model (Fig. 1(K)). As such, we demonstrate proof of principle by showing that the model inversion can correctly identify which model was used to generate the data. Finally, we show that when we change Eq. (23) such that external driving inputs and noise are excluded:

24 $$\begin{aligned}& \begin{aligned}& \frac{d}{dt} ( \operatorname{Im}_{i} ) =- \sum _{j} A_{j} \operatorname{Re}_{j}, \\ & \frac{d}{dt} ( \operatorname{Re}_{i} ) = \sum _{j} A_{j} \operatorname{Im}_{i} \end{aligned} \end{aligned}$$

the Hamiltonian in Eq. (19) is indeed constant in time when we run the model forward with a symmetric intrinsic coupling matrix furnished with the posteriors obtained from Fig. 1(I) (Fig. 1(L)).

### Experimental data

**Figure 2:**
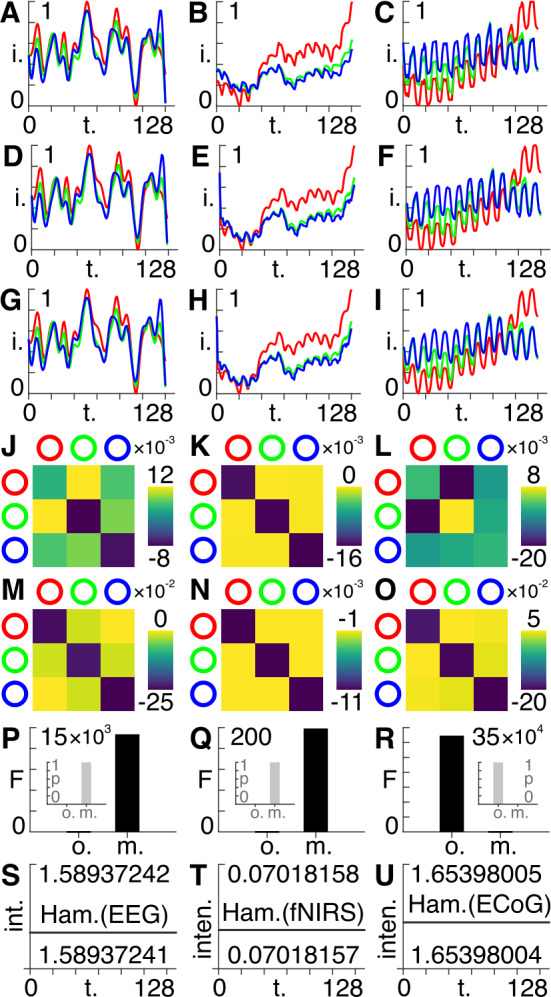
Experimental data. A Normalized intensity (*i*) for the first 128 time points for the first three channels of the EEG data. (**B**) Normalized intensity (*i*) for the first 128 time points for the first three channels of the fNIRS data. (**C**) Normalized intensity (*i*) for the first 128 time points for the first three channels of the ECoG data. (**D**) Estimated timeseries for the EEG data using the original model. (**E**) Estimated timeseries for the fNIRS data using the original model. (**F**) Estimated timeseries for the ECoG data using the original model. (**G**) Estimated timeseries for the EEG data using the modified model. (**H**) Estimated timeseries for the fNIRS data using the modified model. (**I**) Estimated timeseries for the ECoG data using the modified model. (**J**) Posterior estimates of the intrinsic connectivity in the EEG data using the original model. (**K**) Posterior estimates of the intrinsic connectivity in the fNIRS data using the original model. (**L**) Posterior estimates of the intrinsic connectivity in the ECoG data using the original model. (**M**) Posterior estimates of the intrinsic connectivity in the EEG data using the modified model. (**N**) Posterior estimates of the intrinsic connectivity in the fNIRS data using the modified model. (**O**) Posterior estimates of the intrinsic connectivity in the ECoG data using the modified model. (**P**) Approximate lower bound log model evidence given by the free energy (*F*) following Bayesian model inversion for the original (*o*.) and modified (*m*.) models using the EEG data Probabilities (*p*) derived from the log evidence are shown in the inset left. (**Q**) Approximate lower bound log model evidence given by the free energy (*F*) following Bayesian model inversion for the original (*o*.) and modified (*m*.) models using the fNIRS data Probabilities (*p*) derived from the log evidence are shown in the inset left. (**R**) Approximate lower bound log model evidence given by the free energy (*F*) following Bayesian model inversion for the original (*o*.) and modified (*m*.) models using the ECoG data Probabilities (*p*) derived from the log evidence are shown in the inset left. (**S**) The intensity (int.) at every point in time for the non-dissipative form of the modified state equation, i.e., excluding external driving inputs and noise, using the posteriors from the modified EEG model in (**G**) and the Hamiltonian as the observer equation. (**T**) The intensity (inten.) at every point in time for the non-dissipative form of the modified state equation, i.e., excluding external driving inputs and noise, using the posteriors from the modified fNIRS model in (**H**) and the Hamiltonian (Hamil.) as the observer equation. (**U**) The intensity (inten.) at every point in time for the non-dissipative form of the modified state equation, i.e., excluding external driving inputs and noise, using the posteriors from the modified ECoG model in (**I**) and the Hamiltonian (Hamil.) as the observer equation.

Here, we use the same model inversion techniques presented in Fig. 1, except – that instead of synthetic data we use publicly available empirical timeseries collected using EEG [18] (Fig. 2(A)), fNIRS [19] (Fig. 2(B)), and ECoG [20] (Fig. 2(C)) neuroimaging modalities. Instead of the Gaussian function used in Fig. 1, we here use a random external input connected to all three channels, to model ongoing neuronal fluctuations. We also show the estimated timeseries for these datasets using both the original (Fig. 2(D), (E), (F)) and the modified (Fig. 2(G), (H), (I)) models.

Using the same priors as for the synthetic data in Fig. 1, Bayesian model inversion based on the original state equation Eq. (1) furnished posterior estimates of the intrinsic connectivity between the first three channels for the EEG (Fig. 2(J)), fNIRS (Fig. 2(K)), and ECoG (Fig. 2(L)) data. We repeated this procedure by using the modified state equation Eq. (6) to obtain different posterior estimates for the EEG (Fig. 2(M)) fNIRS (Fig. 2(N)), and ECoG (Fig. 2(O)) data. We can then compare the variational free energy – i.e., the model evidence that balances the trade-off between accuracy and complexity – for the original vs. the modified state equation for the EEG (Fig. 2(P)), fNIRS (Fig. 2(Q)), and ECoG (Fig. 2(R)) data, together with the associated probabilities (Figs. 2(P), (Q), (R), insets). In these multimodal examples, the modified (complex, oscillatory) state equation provides a better account (higher variational free energy) for the EEG and fNIRS datasets, but not for the ECoG dataset. Note that the extent of the difference in variational free energies between the original and modified models is determined by the noise parameters in the accompanying DCM code – e.g., the precision and prior variance. Finally, we show that the Hamiltonians are conserved for the non-dissipative cases using the posteriors of the EEG (Fig. 2(S)), FNIRS (Fig. 2(T)), and ECoG (Fig. 2(U)) models.

Both Figs. 1 and 2 can be reproduced in full via the accompanying code.

## Conclusions

The aim of our work was to show how one of the first-order linear equations that dominate computational neuroscience can be rendered compatible with the principle of stationary action with a minimum number of simple modifications. Furthermore, we wanted to show how to embed the Lagrangian model within a data fitting framework in a way that allows it to be readily applied to timeseries of arbitrary dimensionality from any neuroimaging modality.

By making the modifications outlined in this paper, we obtain a state equation that is fundamentally different from the original state equation, with the main difference lying in the solutions to the equations.

To see why this is the case, we can take the simplest case of a single unconnected region in the absence of external driving inputs or noise as modelled by the original (unmodified) state equation:

25 $$\begin{aligned}& \frac{dz}{dt} =z\quad \Rightarrow \quad \int \frac{dz}{z} = \int dt \quad \Rightarrow\quad \ln z=t+c\quad \Rightarrow\quad z=c' e^{t}, \end{aligned}$$

where *c* is a constant of integration and $c'= e^{c}$. We therefore see that the solution increases exponentially in time (or decreases if the original differential equation has a minus sign). Let us now consider the modified state equation for this simple case, as obtained by changing the dependent variable to a complex number and multiplying the left-hand side by the imaginary unit:

26 $$\begin{aligned}& i \frac{dz}{dt} =z\quad \Rightarrow\quad \int \frac{dz}{z} =i \int dt \quad \Rightarrow\quad \ln z=it+ c ' \quad \Rightarrow \quad z=c e^{it} . \end{aligned}$$

The solution now oscillates in time and Eq. (26), unlike Eq. (25), is compatible with the principle of stationary action. We then note that the modified state equation can be reformulated by writing the complex dependent variable *z* in terms of its real *x* and imaginary *y* components such that: $z=x+iy$ and hence:

27 $$\begin{aligned}& i \frac{dz}{dt} =z \quad \Rightarrow\quad i \frac{d}{dt} ( x+iy ) =x+iy, \end{aligned}$$

where we can equate the real and imaginary components to give the following two equations:

28 $$\begin{aligned}& \frac{dy}{dt} =-x,\qquad \frac{dx}{dt} =y \quad \Rightarrow\quad \frac{d^{2} x}{d t^{2}} =-x. \end{aligned}$$

We see that the modified state equation is just another way of representing a simple harmonic oscillator. It should therefore come as no surprise that the modification facilitates compatibility with the principle of stationary action, as harmonic oscillators are readily described by Lagrangian formulations. Furthermore, the fact that the modified form provides a more parsimonious description in some neuroimaging datasets may be indicative of the underlying oscillatory equation capturing the intrinsic oscillations present in neural systems across scales [21].

The imaginary unit *i* famously appears in the Schrödinger equation $i\hslash \dot{\psi } =H\psi $, which we see possesses the same mathematical structure as the computational neuroscience models considered here: it is first order in time and linearly proportional to the dependent variable. One of the reasons Schrödinger introduced the imaginary unit into his equation is that it preserves unitarity, guaranteeing that the value of the integral $\int \psi ^{*} ( \boldsymbol{r},t ) \psi ( \boldsymbol{r},t ) \,d^{3} r$ is independent of time. The value of this integral is the probability that the particle is found somewhere in space and must therefore be conserved on physical grounds (particles do not appear or disappear in non-relativistic quantum theory); the *i* in the Schrödinger equation ensures this conservation mathematically. Every eigenvector component of *ψ* oscillates in time like the function $e^{i\omega t}$, while the corresponding component of $\psi ^{*}$ oscillates like $e^{-i\omega t}$. The conservation of probability follows because $e^{i\omega t} e^{- i\omega t} =1$. On the other hand, we can consider the consequences of calculating a probability in this way when the imaginary unit *i* is not present: $\psi ( t ) \psi ^{*} ( t ) \sim e^{\omega t} e^{\omega t} = e^{2 \omega t} $, i.e. the probability is time dependent and is therefore not conserved.

The preservation of unitarity has potentially interesting consequences regarding neural systems if we are modelling probabilistic quantities such as the chance of observing neural activation beyond a certain threshold. In this case, the presence of the imaginary unit *i* in the modified equations of motion could imply a conservation of neuronal firing or depolarisation in the system, which could in turn be plausibly maintained by the balance between excitation and inhibition [22, 23]. Together with the need for oscillatory solutions, we showed that compatibility between the neuronal state equation and the principle of stationary action necessitated the use of complex variables. A further advantage of working with complex variables is that the adjacency or coupling matrix A can be transformed into a Hermitian form, where the real parts describe dissipation and the complex parts describe oscillatory or solenoidal dynamics [24, 25] that underwrite rhythms in the brain [26] – we refer the reader to the section entitled “Dynamics and statistical physics” in [27] for a comprehensive treatment. More generally, the ability to work with non-dissipative solenoidal dynamics means that one can eschew detailed balance and characterise (neuronal) systems in terms of their nonequilibrium steady states [28–30].

There is an established method for constructing an action for a classical system, often referred to as the MSRDJ (Martin–Siggia–Rose–DeDominicis–Janssen) formalism [31]. This can be applied to arbitrary systems of differential equations of first order but differs from our approach in important ways and addresses different questions. The MSRDJ formalism applies to stochastic differential equations such as the Langevin equation, which include random noise terms (as does the neuronal state equation). Given a set of initial conditions, the solution of a stochastic differential equation depends on the realization of the noise as a function of time, with different realizations producing different solutions. Viewed as a whole, the stochastic differential equation therefore generates a time-evolving probability distribution of solutions, the form of which depends on the nature of the noise. The MSRDJ approach shows how this probability distribution evolves in time and allows one to calculate moments, correlation functions, and other noise-averaged statistical properties as functions of time. The extremum of the MSRDJ action yields the most likely path of the system in the presence of the random noise. It should therefore be stressed that the formalism presented in this paper is not the only way in which a Lagrangian formulation of a first-order linear equation can be obtained – see, for example, the review by Chow et al. [10].

Our approach, by contrast, formulates the complex neuronal state equation in Lagrangian terms, and the corresponding Euler–Lagrange equation reproduces the original differential equation exactly, including the noise terms. When we solve the complex neuronal-state Euler–Lagrange equation, we are therefore evolving a specific solution of the stochastic differential equation, not a probability distribution of solutions. The MSDRJ approach yields access to properties such as the most likely path of escape from a potential well in the sense of large deviations [32–34]. MSDRJ is also useful in terms of finding solutions for particular realizations of the noise – e.g., for comparison with unaveraged experimental timeseries.

The conservation laws uncovered by inspecting the symmetries of the Lagrangian – such as the time-translation invariance that led to the conservation of the value of the Hamiltonian in Eqs. (19), (20), and (21) – are difficult to interpret at this stage, as they have yet to be mapped onto biological mechanisms: this remains a subject for future research to be explored in combination with previous techniques for analysing symmetries in dynamical systems [35]. The purpose of this work was to demonstrate simple techniques by which researchers can create Lagrangian neuronal state models, in a way that allows for symmetries and associated conservation laws to be identified in neural systems.

## Data Availability

All relevant code and data is made available at the following public repository: https://github.com/allavailablepubliccode/Lagrangian.

## Abbreviations

DCM: dynamic causal modelling
DEM: dynamic expectation maximization
EEG: electroencephalography
fNIRS: functional near-infrared spectroscopy
ECoG: electrocorticography
